# Biocompatibility and Angiogenic Effect of Chitosan/Graphene Oxide Hydrogel Scaffolds on EPCs

**DOI:** 10.1155/2021/5594370

**Published:** 2021-05-18

**Authors:** Lifang Zhang, Xinping Li, Congying Shi, Gaoying Ran, Yuting Peng, Shuguang Zeng, Yan He

**Affiliations:** ^1^Department of Oral and Maxillofacial Surgery, Stomatological Hospital, Southern Medical University, 510000 Guangzhou, Guangdong, China; ^2^Clinical Application Center, Guangdong Cord Bank, 510000 Guangzhou, Guangdong, China; ^3^Skeletal Biology Research Center, Department of Oral Maxillofacial Surgery, Harvard School of Dental Medicine, Boston, 02114 MA, USA

## Abstract

Angiogenesis in the field of tissue engineering has attracted significant attention. Graphene oxide has become a promising nanomaterial in tissue engineering for its unique biochemical properties. Therefore, herein, a series of chitosan (CS)/graphene oxide (GO) hydrogel scaffolds were synthesized by crosslinking CS and GO at different concentrations (0.1, 0.5, and 1.0 wt.%) using genipin. Compared with the CS hydrogel scaffolds, the CS/GO hydrogel scaffolds have a better network structure and mechanical strength. Then, we used endothelial progenitor cells (EPCs) extracted from human umbilical cord blood and cocultured these EPCs with the as-prepared scaffolds. The scaffolds with 0.1 and 0.5 wt.%GO showed no considerable cytotoxicity, could promote the proliferation of EPCs and tube formation, and upregulated the expressions of CD34, VEGF, MMP9, and SDF-1 in EPCs compared to the case of the scaffold with 1.0 wt.%GO. This study shows that the addition of graphene oxide improves the structure of chitosan hydrogel and enhances the proliferation activity and angiogenic capacity of EPCs.

## 1. Introduction

Tissue defects caused by trauma, infection, and tumor resection have become a serious problem in healthcare worldwide [1]. Although surgery is the most valid method to treat these defects, it has certain limitations. In this regard, tissue engineering has attracted significant attention. However, as tissue regeneration relies on blood vessels for the transport of nutrients and metabolic wastes, engineered tissues cannot be widely used because they lack a vascular system [2]. Without perfusion from implanted microvasculature, the thickness of engineered tissue constructs in vitro is limited to approximately 200 *μ*m, which is the oxygen diffusional limit [3]. Therefore, sufficient angiogenesis can promote the survival and repair of damaged tissues [4]. Although several studies have been reported on the osteogenesis of scaffold materials, studies on the angiogenesis of these materials are in their infancy at present [5, 6]. Hence, researchers in the field of tissue engineering have started to focus on improving the angiogenesis of tissue-engineered structures [7, 8].

A three-dimensional porous structure is conducive to the growth of endothelial cells and the formation of vascular structures [9, 10]. Hydrogels, as a three-dimensional porous structure, acquire good biocompatibility, degradability, and suitable mechanical properties, and it can be developed and improved to achieve more potential scaffold materials [11, 12]. Chitosan (CS) is a deacetylated product of chitin, which has the advantages of widespread availability and nontoxicity [13]. Hydrogels composed of CS have good biocompatibility and degradability and are widely used as scaffold materials for tissue engineering [14, 15]. However, the weak mechanical strength and low bone conductivity of CS limit the application of these scaffold materials in tissue engineering [16]. To overcome these limitations, many researchers have developed CS composite materials [17, 18] by incorporating inorganic bioactive materials, such as *β*-tricalcium phosphate and hydroxyapatite (HA), using CS to promote bone regeneration [19, 20]. However, the application of these materials is hindered by their insufficient osteoinduction and angiogenesis [21].

In addition, graphene derivatives have gradually become a research hotspot in the field of nanobiomedicine as they can provide a favorable physical and chemical environment for bone formation [22]. Graphene oxide (GO) has shown considerable potential for biomedical applications, including bone tissue engineering, due to its excellent physical and chemical properties and biological activity [23, 24]. GO at an appropriate concentration can induce angiogenesis without significant cytotoxicity [25, 26]. Moreover, GO has abundant oxygen-containing groups, which can increase the deposition of HA [27]. Many researchers have introduced GO into tissue-engineered implants and found that GO is effective in promoting angiogenesis [27–29]. Thus, the addition of GO nanoparticles to the CS hydrogel can overcome the abovementioned limitations of this hydrogel.

In addition to having a suitable three-dimensional scaffold, the growth and proliferation of seed cells are crucial. Endothelial progenitor cells (EPCs), which are precursors of endothelial cells, can stimulate the growth of collateral vessels in ischemic tissues and thus promote angiogenesis [30]. EPCs can be noninvasively obtained from umbilical cord blood and adult peripheral blood, thereby eliminating immunogenicity [31]. Therefore, the angiogenic capacity of a tissue-engineered implant can be enhanced by introducing EPCs into it.

Furthermore, genipin (GNP) is a nontoxic, easily degradable natural substance, which has been widely used for crosslinking biological materials [32]. It was predicted that CS can react with genipin by nucleophilic ring-opening reactions, which is more conducive to the formation of spatial network structures in studies [33, 34]. Several studies have shown that the cytotoxicity of GNP is significantly lower than that of the classical crosslinking agent glutaraldehyde in vitro [35], and GNP has been continuously applied to manufacture biopolymer scaffolds in bone tissue engineering [36, 37].

Accordingly, in this study, a series of CS/GO hydrogel scaffolds were fabricated by crosslinking CS and GO at different concentrations using GNP. These hydrogel scaffolds exhibited significantly improved stability. Subsequently, we investigated the physical and chemical properties of these scaffolds. Moreover, the angiogenic potential of these scaffolds was evaluated by coculturing them with human EPCs, and the possible mechanisms have been discussed.

## 2. Materials and Methods

### 2.1. Hydrogel Production

Herein, 2.0 g CS (degree of deacetylation ≥ 95%, Macklin, Shanghai, China) was dissolved in 100 mL aqueous hydrochloric acid (0.1 mol/L, Sigma-Aldrich, St. Louis, State of Missouri, USA) under sterile conditions. The pH of the resulting solution was adjusted to approximately 7.25 by *β*-glycerophosphate sodium solution (70% *w*/*v*, Solarbio, Beijing, China). Then, appropriate amounts of GNP solution (1% *w*/*v*, Sigma-Aldrich) and GO aqueous solution (3 mg/mL, Macklin) were added to the abovementioned solution. By mechanical stirring and ultrasonic breaking, CS/GO mixed solutions with different GO concentrations were prepared. Thereafter, these mixed solutions were centrifuged at 4°C and 3000 r/min for 10 min and then placed in a biochemical incubator at 37°C to form the hydrogels. Finally, these hydrogels were frozen at -80°C for 4 h and then placed in a vacuum freeze dryer (Biobase, Jinan, Shandong province, China) to construct hydrogel scaffolds.

### 2.2. Scanning Electron Microscopy (SEM)

All lyophilized hydrogel scaffolds were cut into cubes with a side length of 0.5 cm followed by sputter coating with gold in liquid nitrogen. The internal structure and morphology of the scaffold sections were examined by SEM (Hitachi S-3700N, Chiyoda, Tokyo). Three samples of each group were chosen, and pictures were taken under an electron microscope. Three areas of each sample were selected randomly and measured by ImageJ. Typically, the average pore size was calculated.

### 2.3. Fourier Transform Infrared Spectroscopy (FTIR)

Hydrogel scaffolds in appropriate amounts were ground and mixed with KBr (Macklin) particles in a 1 : 300 ratio. Then, they were scanned 64 times using a Fourier transform infrared spectrometer (TENSOR 27, Bruker, Karlsruhe, Baden-Wurttemberg, Germany) in the range of 4000–400 cm^−1^ at a resolution of 2 cm^−1^, and Fourier transform infrared spectra were recorded.

### 2.4. X-Ray Powder Diffraction (XRD)

The abovementioned samples were analyzed using an X-ray diffractometer (D8X, Bruker), and the corresponding XRD patterns were acquired. The scanning area was in the 2*θ* range of 0–40°, and the scanning speed was 2°/min.

### 2.5. Tensile Properties

Lyophilized scaffolds were cut into 2 cm × 0.5 cm strips, and their tensile properties were measured using an electronic universal material testing machine (Qingji Instrument Technology Co., Ltd, Shanghai, China) and a 100 N load cell at a loading rate of 10 mm/min. The measurement was performed five times, and the average value was calculated.

### 2.6. Swelling Rate (SR)

Suitable amounts of lyophilized scaffolds were weighed, immersed in a large amount of ultrapure water at 25°C for 24 h, and then weighed again after removing the surface moisture:

(1) $$\begin{array}{c} \text{SR}=100\%\times \frac{\text{Ws}-\text{Wd}}{\text{Wd}}, \end{array}$$

where Ws and Wd are the weights of the wet and dry hydrogel scaffolds, respectively.

### 2.7. Porosity (*ε*) Test

The specific method was as follows [38]: hydrogels (dry mass M0) were immersed in ethanol in a weighing bottle. The bottle was weighed before (Ma) and after (Mb) removing the wet scaffold. Meanwhile, a 50 mL pycnometer filled with ethanol was weighed with the weight denoted as M1. The ethanol was poured out, and the wet scaffold previously soaked in ethanol was placed in the pycnometer, and ethanol was added until the pycnometer was filled to the same mark. The pycnometer was weighed again with weight denoted as M2. Porosity was calculated according to the following equations (*ρ* is the density of ethanol):

(2) $$\begin{array}{c} \text{V}1=\left(\text{Ma}-\text{Mb}-\text{M}0\right)/\rho ,\\ \text{V}2=\left[\left(\text{Ma}-\text{Mb}\right)-\left(\text{M}2-\text{M}1\right)\right]/\rho ,\\ \varepsilon =\left[\text{V}1/\text{V}2\right]\times 100\%=\left(\text{Ma}-\text{Mb}-\text{M}0\right)/\left[\left(\text{Ma}-\text{Mb}\right)-\left(\text{M}2-\text{M}1\right)\right]\times 100\%, \end{array}$$

where V1 is the volume of scaffold pores, V2 is the apparent volume of the scaffold, and *ε* is the porosity of the scaffold. Measurements were repeated three times for the same sample, and the average value was obtained.

### 2.8. Cell Subculture

#### 2.8.1. Subculture of EPCs

After the EPCs (purchased from the Guangdong Cord Blood Bank, Guangzhou, Guangdong province, China) adhere to the wall, the medium was changed every two days. The cells can be subcultured when they have grown to 80%. After removing the original medium, rinse with PBS (Thermo Fisher Scientific, Waltham, Massachusetts, USA) twice and add 0.5 mL 0.25% trypsin (Sigma-Aldrich). When the cells are round, add medium to stop the digestion. After centrifugation at 800 rpm for 3 min, resuspension with Endothelial Cell Growth Medium (EGM) was used to inoculate in a culture flask and then cultured in a 37°C, 5% CO_2_ incubator (Thermo Fisher Scientific).

### 2.9. Cell Identification

Herein, 1 × 10^6^ EPCs were collected, and fluorescent dyes were used to conjugate cell surface markers: CD45/FITC, CD14/PE, CD34/PE, CD133/PE, CD31/FITC, CD105/FITC, and KDR-PE (Thermo Fisher Scientific). Cells were fixed with 4% paraformaldehyde (Beyotime Biotechnology). After being stained and washed with flow cytometry staining buffer (PBS + 1%BSA + 0.1%NaN_3_), the cells were analyzed using a flow cytometer (BD Biosciences, Franklin Lakes, New Jersey, USA).

### 2.10. Tube Formation Experiment

Matrigel (Corning, New York, USA) was melted at 4°C and added to a 96-well plate precooled at -20°C followed by the removal of air bubbles. Then, this plate was placed in a cell incubator at 37°C for 1 h. The EPCs were treated with serum-free Endothelial Cell Growth Medium (EGM) for 1 h and then placed on a 96-well plate covered with Matrigel at 5 × 10^4^ cells/well; thereafter, this plate was placed in a 37°C, 5% CO_2_ humidified incubator. After 8 h, tube formation was observed using a microscope (Olympus IX51, Japan), and images were acquired.

### 2.11. Cocultivation and Grouping of EPCs and Hydrogel Scaffolds

Hydrogel scaffolds were placed in a well plate, and then, an appropriate amount of EPCs was inoculated into these scaffolds according to the experimental requirements. Based on the concentration of GO in the scaffolds, the coculture system was divided into five groups: EPCs, CS; EPCs, CS/0.1 wt.%GO; EPCs, CS/0.5 wt.%GO; EPCs, CS/1.0 wt.%GO; and EPCs.

### 2.12. Cell Counting Kit-8 (CCK-8) Assay

Scaffolds in appropriate amounts were fabricated under aseptic conditions and spread on a 24-well plate; then, 500 mL of a medium containing 5 × 10^4^ EPCs was seeded in each well, and 50 *μ*L CCK-8 solution (Dojindo, Kumamoto, Japan) was added to each well every 36 h. After incubation at 37°C for 2 h, 100 *μ*L medium was withdrawn and added to a 96-well plate for absorbance measurement at 450 nm using a microplate reader (Bio-Rad, Hercules, California, USA). In each test, three duplicate wells were set for all specimens.

### 2.13. Lactate Dehydrogenase (LDH) Toxicity Assay

Scaffolds were synthesized under aseptic conditions and spread in 96-well plates. The EPCs were digested and seeded at 1 × 10^4^ cells/well. After 1, 3, and 5 days, three wells of cells for each group were centrifuged at 400 g for 5 min, and the supernatants were obtained. LDH working solution (Beyotime Biotechnology) was prepared according to the instructions of the LDH. The LDH working solution was completely mixed with the test solution. Approximately 120 *μ*L of supernatant was withdrawn from each well and put in a new 96-well plate. At room temperature, the 96-well plate wrapped with aluminum foil was placed on a horizontal shaker for slow shaking followed by incubation in the dark for 30 min. Then, the absorbance was measured at 490 nm using the abovementioned microplate reader.

### 2.14. Live/Dead Staining

The EPCs were seeded on 12-well plates at 5 × 10^5^ cells/well. After culturing in a cell incubator, the culture solution was withdrawn and gently soaked three times with PBS to ensure the removal of the active esterase present in the culture medium.

#### 2.14.1. Configuration of the Staining Working Solution (Beyotime Biotechnology)

Calcein and propidium iodide were equilibrated at room temperature for 30 min, and the staining working solution was configured according to the instructions. The staining working solution (200 *μ*L) was added to each well to cover the bottom of the well plate, followed by incubation in the dark for 30 min and gentle washing three times with PBS for 5 min each time. The green and red fluorescence of the same region was examined using a fluorescence microscope (Olympus IX51, Japan).

#### 2.14.2. Measurement of the Number of Dead and Live Cells

Trypsin without ethylenediaminetetraacetic acid was used to digest the previously seeded and soaked EPCs, followed by centrifugation and washing twice with PBS to resuspend the cells. Then, the cells of each group were stained by the abovementioned process followed by washing and centrifugation with PBS. Finally, the measurement was conducted using the flow cytometer.

### 2.15. Tube Formation Assay

Cells were inoculated according to the instructions provided on the CCK-8 kit and continuously cultured for 7 days. The Matrigel basement membrane matrix (BD Biosciences, Franklin Lakes, New Jersey, USA) was thawed at 4°C, mixed with the basal medium in equal proportion, pipetted into precooled 96-well plates, and incubated at 37°C for 30 min. After Matrigel polymerization, EPCs were treated with serum-free EGM for 1 h and then digested and reseeded on Matrigel in 96-well plates at 1 × 10^4^ cells/well. After being incubated at 37°C for 8 h, the cells were imaged using a microscope (Leica, Wetzlar, Hesse-Darmstadt, Germany), and the images were analyzed and interpreted using ImageJ software.

### 2.16. Quantitative Reverse Transcription Polymerase Chain Reaction (qRT-PCR)

The EPCs were seeded in 6-well plates at 1 × 10^6^ cells/well and continuously cultured for 7 days. Total RNAs of the cells of each group were extracted with the TRIzol reagent (Sigma, Missouri, USA) and reverse transcribed into cDNA using an Evo M-MLV RT Premix kit (Accurate Biology, Changsha, Hunan, China). The SYBR® Green Premix Pro Taq HS qPCR Kit (Accurate Biology, Changsha, Hunan, China) was used for PCR amplification, and the total volume of the reaction system was 20 *μ*L. PCR was performed employing the ABI PRISM® 7500 sequence detection system. The reaction conditions were as follows: 95°C for 30 s, followed by 40 cycles of 95°C for 5 s and 60°C for 30 s. Thereafter, the 2-*ΔΔ*CT method was used to calculate the relative DNA expression. All primers used in this experiment were synthesized by TsingKe (Beijing, China) and are listed in Table 1.

### 2.17. Western Blot Analysis

Cells were seeded and cultured as mentioned in Section 2.15. The cells were lysed with RIPA Lysis Buffer (Sigma, Missouri, USA), placed on ice for 30 min, and then centrifuged at 12000 g for 15 min at 4°C, and the supernatant was aspirated. The bicinchoninic acid protein kit (China Biyuntian Biotechnology Co., Ltd) was used to determine the protein concentration of cells. Sodium dodecylsulphate-polyacrylamide gel electrophoresis (SDS-PAGE) loading buffer (YongJin, Guangzhou, Guangdong, China) was added to the cells in a 4 : 1 ratio followed by heating at 100°C for 10 min. The sample (20 *μ*g) was subjected to SDS-PAGE and then transferred to a polyvinylidene difluoride membrane (Millipore, Billerica, MA, USA), which was then blocked with 5% skim milk at room temperature for 2 h. The resulting membrane was incubated with the primary antibody at 4°C overnight and then with the specific secondary antibody at room temperature for 2 h. Thereafter, ChemiDoc XRS+ (Bio-Rad) was employed to detect the protein band by chemiluminescence, and the images were analyzed using the Quantity One software. The following antibodies were used: CD34 (1 : 1000; Proteintech, 3C8G12), VEGF (1 : 1000; Thermo Fisher, MA5-13182), SDF-1 (1 : 1000; Abcam, ab9797), MMP9 (1 : 3000; Thermo Fisher, PA5-83748), and GAPDH (1 : 5000; Fude Biotechnology, FD0063-100).

### 2.18. Statistical Analysis

Statistical analyses were carried out using SPSS 22.0 software. All experimental data were expressed as the mean difference ± standard deviation. Comparisons between different groups were conducted using an independent sample *t*-test. Moreover, for multiple specimens, initially, the homogeneity of variance was calculated by Levene's test. If the variance was uniform, one-way analysis of variance (ANOVA) was simultaneously performed using SNK multiple comparisons between groups; if the variance was uneven, Welch's robust ANOVA was conducted, and Dunnett's T3 test was employed for multiple comparisons between groups. *P* < 0.05 was considered statistically significant.

## 3. Results

### 3.1. Features and Structural Characterization of CS/GO Hydrogel Scaffolds

**Figure 1:**
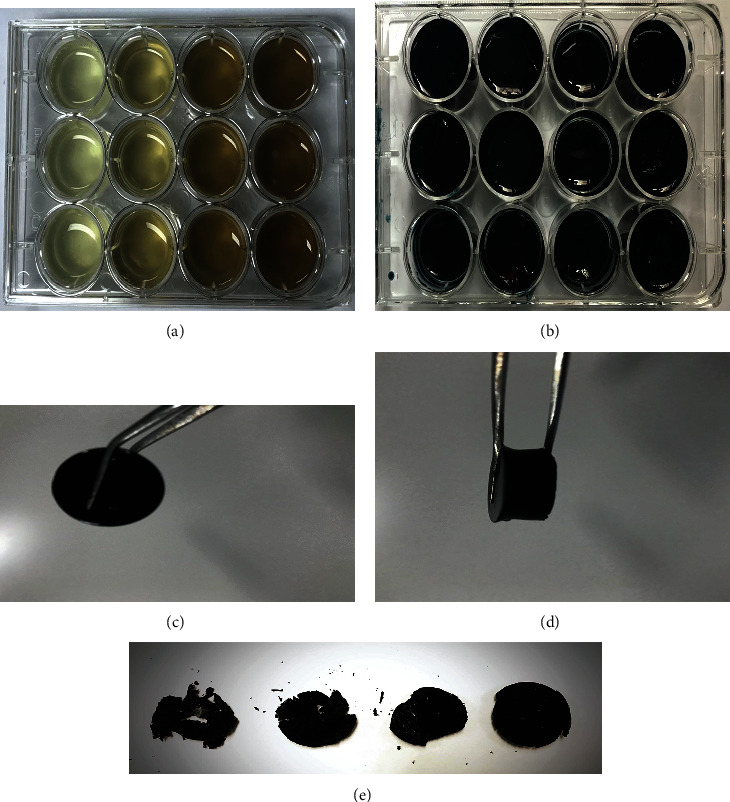
Features of CS/GO hydrogel scaffolds (from left to right: the hydrogel scaffolds with 0, 0.1, 0.5, and 1.0 wt.%GO): (a) CS/GO hydrogel scaffolds directly spread on a 12-well plate. (b) CS/GO hydrogel scaffolds crosslinked for 24 h. (c) Front view of the hydrogel scaffolds. (d) Side view of the hydrogel scaffolds. (e) CS/GO hydrogel scaffolds after vacuum freeze-drying.

The as-prepared CS/GO hydrogel scaffolds were dark blue, jelly-like, and elastic (as observed by the naked eye), and their internal structure was loose, which recovered after deformation under light pressure (Figures 1(a)–1(d)). After vacuum freeze-drying, their texture became hard and brittle, and the pure CS hydrogel scaffold was not suitable for clamping and had low mechanical strength (Figure 1(e)).

**Figure 2:**
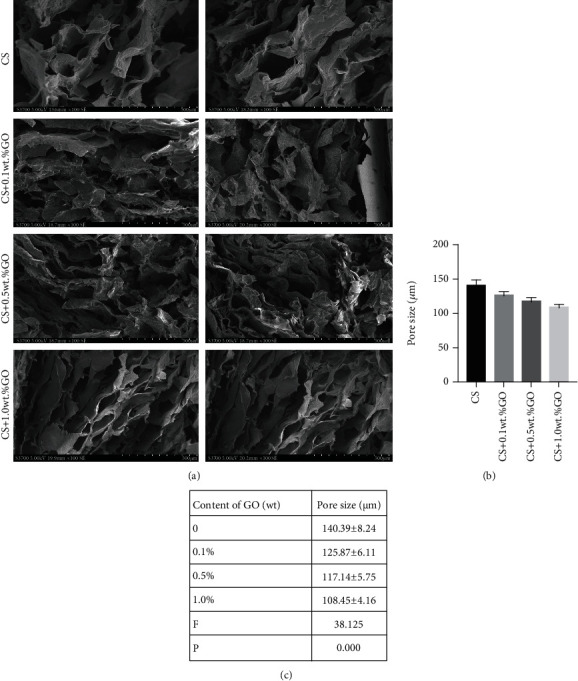
SEM images of the CS/GO hydrogel scaffolds. (a) SEM image of the cross-section of the scaffolds of each group (bar = 500 *μ*m). (b, c) Aperture size of each group randomly measured by ImageJ. ^∗^*P* < 0.05 compared with the cases of other groups.

The structure of the pure CS hydrogel scaffold is disordered and irregularly curled. With an increase in the GO concentration, the internal shape of this scaffold slowly became uniform, and the irregular curling gradually improved (Figure 2(a)), indicating that the addition of GO improved the internal structure of the CS hydrogel scaffold. SEM images also show that all the hydrogel scaffolds are highly porous and interconnected, with pore size in the range of 100–150 *μ*m (Figures 2(b) and 2(c)).

**Figure 3:**
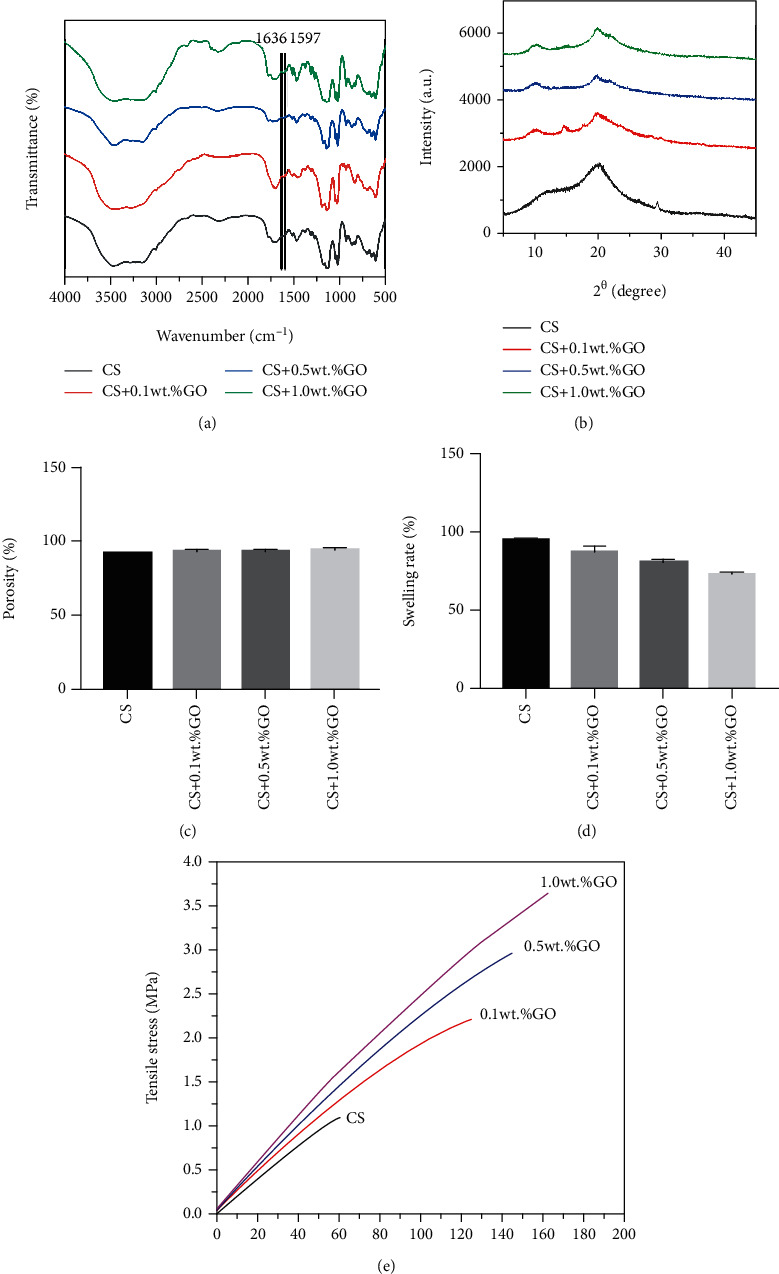
Physical and chemical properties of CS/GO hydrogel scaffolds. (a) FTIR spectra, (b) XRD patterns, and (c) porosity of the hydrogel scaffolds of each group (^∗^*P* > 0.05). (d) Swelling rate of the hydrogel scaffolds of each group (^∗^*P* < 0.05). (e) Tensile properties of the hydrogel scaffolds of each group.

We used a series of experiments to further characterize the physical and chemical properties of the CS/GO hydrogel scaffolds. FTIR spectra of the CS hydrogel scaffold exhibited two characteristic absorption bands at 1636 and 1597 cm-1, corresponding to the C-O tensile vibration of the acetylated amino group (-NHCO-) and the N-H bending of -NH_2_, respectively. In the case of the CS/GO hydrogel scaffolds, the -NH_2_ absorption band shifted to a lower value, whereas the intensity of the -NHCO- band increased; this may be because the -NH_2_ group of CS reacts with the -COOH group of GO, leading to the formation of an -NHCO- graft point (Figure 3(a)).

XRD patterns of each group of scaffolds showed the characteristic peak of CS at 2*θ* = 20.1°, which indicated the crystallinity of CS (Figure 3(b)). With an increase in the GO concentration, the intensity of this peak gradually decreased, and a new sharp peak appeared near 2*θ* = 11.0°, which became more evident with an increase in the GO concentration. All these results suggest that the introduction of GO indeed changes the crystal structure of CS and is consistent with the FTIR results.

### 3.2. Physical and Chemical Properties of the CS/GO Hydrogel Scaffolds

The porosity test indicated that the porosity of each group of scaffolds was ranged from 92% to 95%. However, it slowly increased with an increase in the concentration of GO from 0.1 to 1.0 wt.% (Figure 3(c)). There are no differences in porosity among each group (*P* = 0.095 > 0.05).

As shown in Figure 3(c), the CS hydrogel scaffold has a higher SR than that of the CS/GO hydrogel scaffolds, and the SR of the CS/GO hydrogel scaffolds gradually decreased with an increase in the GO concentration from 0.1 to 1.0 wt.%.

To further explore the influence of GO on the properties of the CS hydrogel scaffold, the tensile properties of the CS/GO hydrogel scaffolds were investigated. The tensile stress-strain curves for each group of scaffolds are shown in Figure 3(e), and the mechanical properties are summarized in Table 2. Results suggest that when the GO concentration was increased from 0 to 1.0 wt.%, the elastic modulus, tensile strength, and elongation at break increased from 0.829 to 22.026 MPa, from 1.363 to 7.153 MPa, and from 61.3 to 161.5%, respectively.

### 3.3. Identification of EPCs

**Figure 4:**
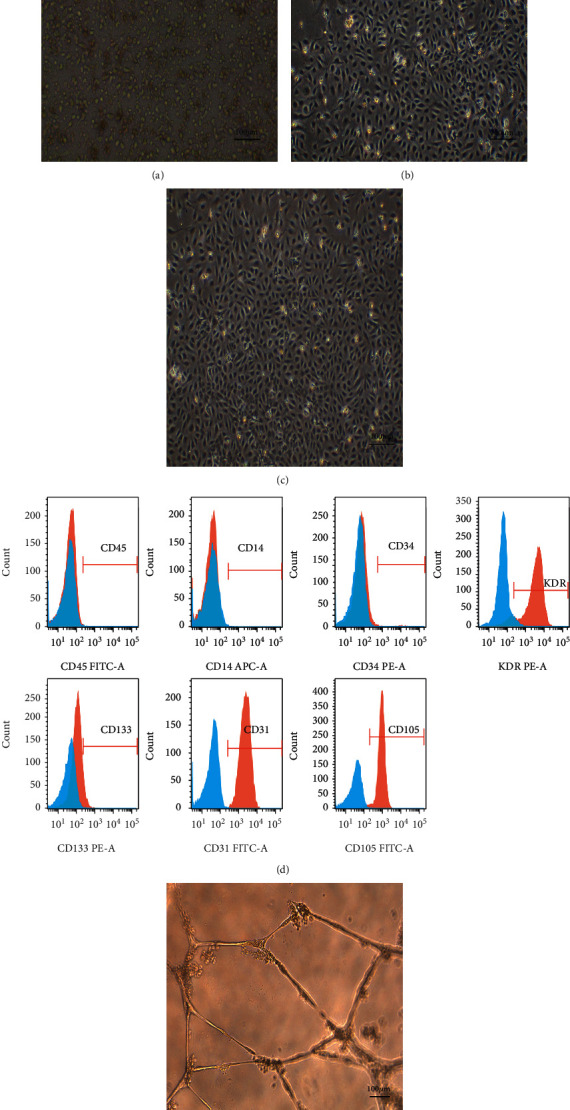
Isolation, culture, and identification of EPCs (provided by Guangdong Cord Blood Bank). Newly isolated primary cells (a) and cells cultured for 3–5 (b) and 7 (c) days. (d) Flow cytometry identification results of primary cells. (e) Matrigel tubule formation experiment of primary cells.

To verify the hypothesis that the CS/GO hydrogel scaffold can improve the angiogenesis of cells, we used EPCs from human umbilical cord blood as seed cells for investigation. The freshly isolated primary cells were round (Figure 4(a)). After being cultured for 3–5 days, the cells proliferated, grew into nearly spindle-shaped cells, and demonstrated “colony-like growth” (Figure 4(b)). After 7 days, the cells rapidly proliferated and were connected to each other in a typical “paving stone” pattern (Figure 4(c)). Flow cytometry results showed that the third-generation EPCs had low expressions of CD45 (0.11%), CD14 (0.21%), CD34 (4.21%), and CD133 (1.16%) and high expressions of the endothelial cell markers CD31 (99.76%), CD105 (98.45%), and KDR (92.55%) (Figure 4(d)). The results of the tubule formation experiments suggested that the cells connected end-to-end during incubation with Matrigel to form a tubule-like structure, and each tubule was connected to form a tube-like structure similar to that of the vascular lumen (Figure 4(e)). These results confirm that the EPCs extracted in this experiment are a class of progenitor cells that can differentiate into endothelial cells and have the ability to form tubules.

### 3.4. Evaluation of the Effect of CS/GO Hydrogel Scaffolds on the Proliferation of EPCs and Cytotoxicity of These Scaffolds

**Figure 5:**
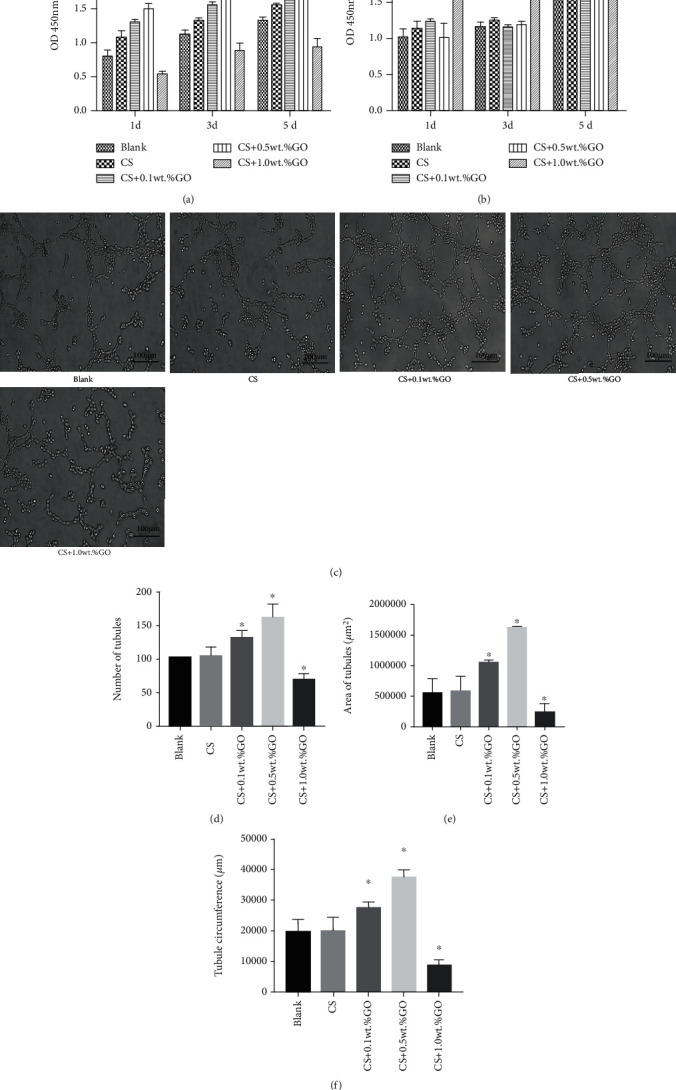
Effect of CS/GO hydrogel scaffolds on the proliferation and angiogenesis of EPCs. (a) Proliferation level of cells in each group determined by the CCK-8 assay. (b) Cytotoxicity of hydrogel scaffolds in each group evaluated by the LDH assay. (c–f) Angiogenesis ability of cells in each group determined using tube formation. ^∗^*P* < 0.05.

During the coculture of the hydrogel scaffolds with EPCs, we explored the effect of the CS/GO hydrogel scaffolds on the proliferation of EPCs and the cytotoxicity of these scaffolds (Figures 5(a) and 5(b)). First, the proliferation of the EPCs in the five culture groups was analyzed by the CCK-8 assay on 1, 3, and 5 days after culture. Cell proliferation was considerably inhibited when the concentration of GO was increased to 1.0 wt.%, whereas it was promoted when the concentration of GO was increased from 0 to 0.5 wt.%. The LDH toxicity assay again confirmed that the group containing 1.0 wt.%GO indeed had clear cytotoxicity, which indicated that GO over a certain concentration caused cytotoxicity.

**Figure 6:**
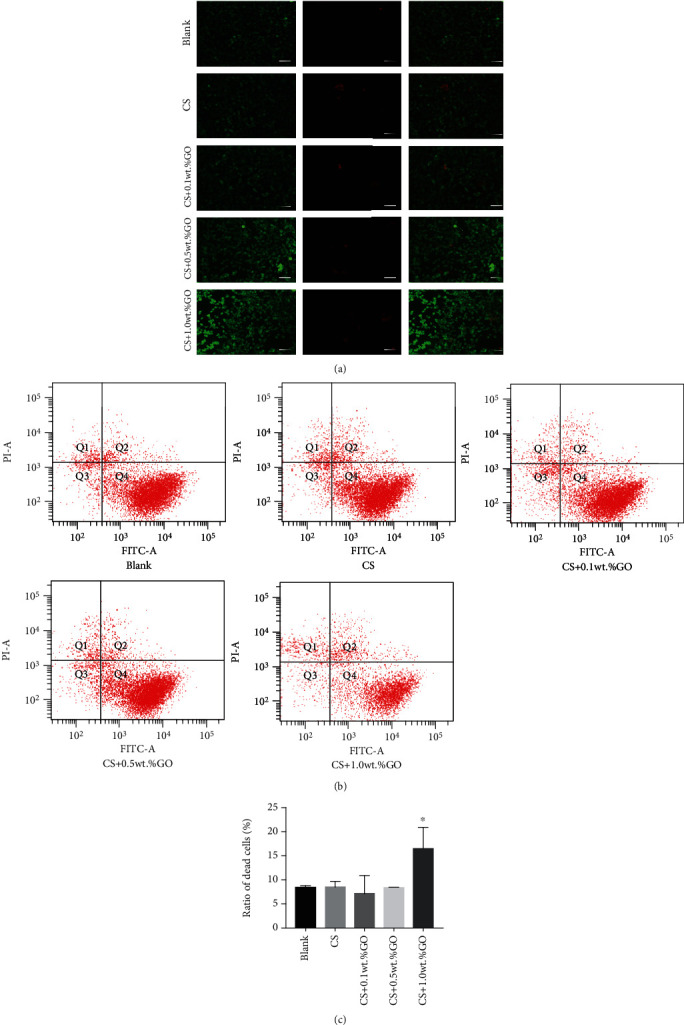
Dead and live cell staining of the EPCs in each coculture group: (a) staining images obtained using the fluorescence microscope; (b) dead and live cell staining results acquired by FCM; (c) ratio of dead cells in each group. ^∗^*P* < 0.05.

To further confirm the cytotoxicity of the hydrogel scaffold with 1.0 wt.%GO, a live/dead staining was performed, and images were obtained using the fluorescence microscope, and the numbers of dead and live cells were recorded by flow cytometry (Figure 6). The proportion of dead cells in the EPCs, CS; EPCs, CS/0.1 wt.%GO; EPCs, CS/0.5 wt.%GO; EPCs, CS/1.0 wt.%GO; and EPC groups was less than 10% (*P* < 0.05, compared with the case of the EPCs, CS/1.0 wt.%GO group), and there was no statistical difference in the proportion of dead cells between the EPCs; EPCs, CS; EPCs, CS/0.1 wt.%GO; and EPCs, CS/0.5 wt.%GO groups (*P* > 0.05). In the EPCs, CS/1.0 wt.%GO group, the proportion of dead cells was up to 16.5% (*P* < 0.05, compared with the cases of other groups). All these results clearly confirm that the hydrogel scaffold with 1.0 wt.%GO has substantial cytotoxicity.

### 3.5. Effect of the CS/GO Hydrogel Scaffold on the Angiogenesis of EPCs

Results of the tube formation analysis (Figures 5(c)–5(f)) demonstrated that on the seventh day of coculture, the EPCs in the EPCs, CS/0.5 wt.%GO group had most significant tube formation ability, whereas those in the EPCs, CS/1.0 wt.%GO group had the lowest tube formation ability (*P* < 0.05). Moreover, the tube formation abilities of EPCs in the EPCs and EPCs, CS groups were not significant (*^Δ^P* > 0.05).

**Figure 7:**
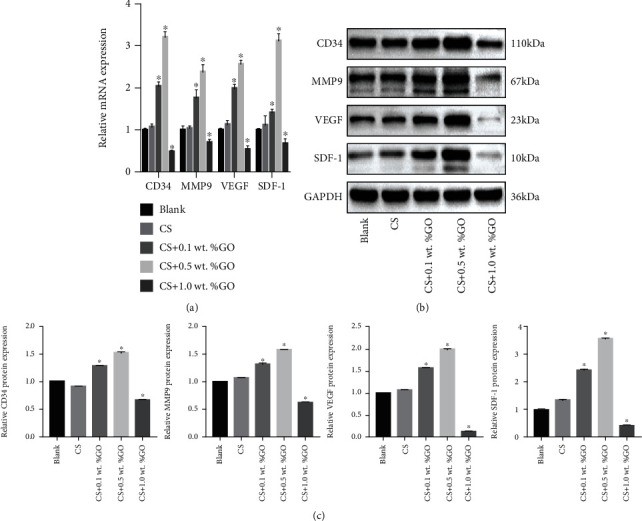
(a) qRT-PCR analysis of the CD34, VEGF, MMP9, and SDF-1 expressions in each group. (b) Western blot analysis results of CD34, VEGF, MMP9, and SDF-1 protein expressions in each group. ^∗^*P* < 0.05 compared with the blank group.

To further study the role of CS/GO hydrogel scaffolds in promoting angiogenesis, qRT-PCR and Western blot analysis were conducted, and the results showed that on the seventh day of coculture, the expressions of CD34, VEGF, MMP9, and SDF-1 in EPCs were considerably upregulated with an increase in the GO concentration when compared with the case of the EPC group. However, the expressions of CD34, VEGF, MMP9, and SDF-1 were inhibited in the EPCs, CS/1.0 wt.%GO group (Figure 7). As shown in Figure 7(a), the relative mRNA expressions of the EPCs, CS and EPC groups were not significant (*P* > 0.05), whereas those of the other groups had statistical significance in multiple comparisons (^∗^*P* < 0.05, compared with that of the EPC group). The results of Western blot analysis are similar to those of the qRT-PCR (Figures 7(b) and 7(c)). All these results indicate that in an appropriate concentration range, GO can promote the proliferation and angiogenic ability of EPCs, which will contribute to improving angiogenesis in tissue engineering.

## 4. Discussion

In 1993, tissue engineering was proposed by American scholars and subsequently brought a new dawn to the reconstruction and repair of tissue defects [39]. Tissue engineering is a discipline that covers multidisciplinary fields such as biology, medicine, and engineering. It develops new biological materials to develop biological substitutes that can repair or improve defective tissues, organs, or parts of the human body [40, 41]. Although the existing biomaterials have made some progress in the repair of tissue defects, the angiogenesis of regenerated tissue is still a research problem. Although currently different studies promote tissue regeneration through angiogenesis, none of the methods have been successfully applied clinically [5, 8, 42]. Therefore, continuous exploration and development of scaffold materials that promote tissue angiogenesis and the success rate of tissue defect repair have important medical value.

GO was brought into tissue engineering for its good mechanical properties and angiogenesis properties [43, 44]. So, we added GO into chitosan hydrogel in order to make an ideal hydrogel scaffold material. A single biomaterial has limitations due to its own performance. Therefore, the composite materials have their superior performance due to multiple materials being mutually reconstructed and will play a vital role in tissue engineering scaffolds to repair tissue defects. The application of crosslinking and covalent bonding significantly improves the stability of the hydrogel [45, 46]. So, genipin was used as the crosslinking agent in this study. Accordingly, a series of CS/GO hydrogel scaffolds were fabricated by crosslinking CS and GO at different concentrations using GNP in our research.

Analyzing the features and structural characterization of CS/GO hydrogel scaffolds, we attained the conclusions as follows. With an increase in the GO concentration, the strength of the scaffold gradually increased. Therefore, the addition of GO to the CS hydrogel scaffold is effective in increasing the hardness of this scaffold. Furthermore, the pore size of the scaffold slowly decreased with an increase in the concentration of GO. This further showed that the addition of GO improved the structure of the CS hydrogel scaffold, making the spatial network structure inside the scaffold more uniform and porous.

**Figure 8:**
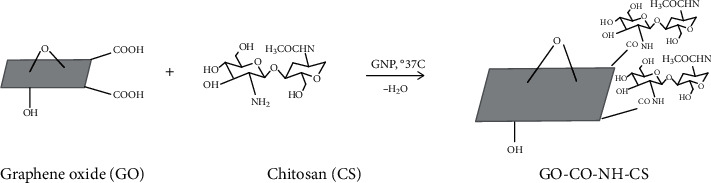


We used a series of experiments to further characterize the physical and chemical properties of the CS/GO hydrogel scaffolds. From the FTIR and XRD, the introduction of GO indeed changed the crystal structure of CS. We predicted a possible schematic diagram of the material structure in the Figure 8.

The porosity test simply confirms that the introduction of GO increases the porosity of the CS hydrogel scaffold. Furthermore, a better porosity can promote cell spreading and facilitate cell proliferation [47, 48]. In addition, SEM images show that the introduction of GO makes the structure of the CS hydrogel scaffold more uniform, reduces the pore size, and increases the porosity. Considering that the porosity increased and the structure of the hydrogel became more uniform by adding GO, it is attributed to physical crosslinking between -COOH and -OH of GO and -NH_2_ of CS, which may even chemically crosslink upon the addition of GNP. Hence, we predict that the cells can expand and grow better in hydrogel scaffold materials, which would facilitate the exchange of substances between the implanted body and the outside environment for providing more energy to promote the formation of blood vessels and tissues. All these results suggest that the CS/GO hydrogel scaffold may become a potential scaffold for tissue engineering.

This implied that the presence of GO enhanced the swelling rate of the CS hydrogel scaffold possibly owing to physical crosslinking between -COOH and -OH of GO and -NH_2_ of CS, which may even chemically crosslink upon the addition of GNP; however, this requires further confirmation. Thus, it can be speculated that crosslinking inhibits the swelling of the CS hydrogel scaffold and thereby reduces the SR.

Therefore, the addition of GO significantly improved the tensile properties of the CS hydrogel scaffold. This is consistent with the previously reported results of CS/GO hydrogel films with different GO concentrations [49, 50]. In summary, we found that the addition of GO changed the structure of the chitosan hydrogel, which affected the physical and chemical properties of the material.

To further explore the biocompatibility and angiogenic effect of chitosan/graphene oxide hydrogel scaffolds, the EPCs were chosen. ECs (endothelial cells) cannot be widely used for the vascularization of bone tissue because of their low proliferation and low utilization [51]. Therefore, we used EPCs as the seed cells. The CCK-8 and cytotoxicity assay results showed that when the concentration of GO was higher than 0.5 wt.%, it inhibited the proliferation of EPCs and caused significant cytotoxicity to the cells. This is consistent with the results reported by Wang et al.; that is, GO has no considerable cytotoxicity at a suitable concentration, whereas it exhibits substantial cytotoxicity at doses higher than 50 *μ*g/mL [52]. Therefore, as a new biomaterial, GO at an appropriate concentration can be used to prepare tissue engineering scaffold materials.

During the tube formation experiment, the CS hydrogel scaffold did not significantly promote angiogenesis, whereas after the addition of GO, it stimulated tube formation. However, when the concentration of GO was up to 1.0 wt.%, tube formation was inhibited. This proves that high concentrations of GO may inhibit angiogenesis of the EPCs. To further explore the molecular mechanism of blood vessel formation by the CS/GO hydrogel scaffolds, we conducted more experiments. CD34, VEGF, MMP9, and SDF-1 were chosen to explore the mechanism.

CD34 is a transmembrane glycoprotein that is selectively expressed on the surface of EPCs. SDF-1 and VEGF play a key role in EPC function. Studies have shown that SDF-1 promotes the expression of VEGF in ECs and is an important cytokine to mobilize EPC [53]. Wang et al. confirmed that metallothionein (MT) promotes the angiogenesis of EPCs mediated by the HIF-1*α*/SDF-1/VEGF pathway [54]. Several studies proved that the SDF-1/VEGF pathway is vital to the angiogenesis of EPCs [55, 56]. In our research, the expressions of the angiogenesis-related genes CD34, VEGF, MMP9, and SDF-1 were examined and found to be upregulated at an appropriate concentration of GO. Western blot analysis results also confirmed this phenomenon. Thus, we concluded that GO may regulate the angiogenic ability of EPC through the SDF-1/VEGF signaling pathway. We found few studies about the angiogenic mechanism of GO on EPCs, while different from ECs. Mukherjee et al. [26] found that the activation of phospho-eNOS and phospho-Akt might be the plausible mechanisms for GO- and rGO-induced angiogenesis of HUVECs. From the research, we concluded that the immune microenvironment induced by GO at an appropriate concentration promotes the angiogenesis of HUVEC through the VEGF pathway [57]. However, further investigation is required to understand the corresponding specific signaling molecular mechanism.

We predict that the suitable concentration of CS/GO hydrogel scaffolds can promote the proliferation and tube formation of EPCs and further stimulate the formation of blood vessels. The long-term prospect of this research is providing little evidence for the role of CS/GO hydrogel scaffolds combined with EPCs in promoting angiogenesis and the repair of tissue defects. We hope that these findings can provide a new theoretical basis for the application of the combination of CS/GO hydrogel scaffolds and EPCs in promoting tissue engineering repair and in tissue regenerative medicine. However, our research has several limitations. First, we did not comprehensively study the specific mechanism of CS and GO crosslinking by GNP. Second, only a few detailed studies were conducted on the molecular mechanism of EPC regulation by the CS/GO hydrogel scaffold. Third, as in vivo experiments were not performed, we cannot verify the role of this scaffold material in repairing bone defects in vivo.

## 5. Conclusion

In this study, we successfully prepared a series of CS/GO hydrogel scaffolds with different concentrations of GO. The addition of GO improved the structure of the CS hydrogel scaffold, facilitated the formation of the spatial network structure, and improved the physical, chemical, and mechanical properties of the scaffold. At an appropriate concentration, GO had no significant cytotoxicity and promoted the extension and proliferation of EPCs, thereby stimulating the formation of blood vessels. However, research on the role of GO in basic and clinical medical fields is still in its infancy, and the molecular mechanism involved in angiogenesis needs further investigation. In conclusion, our study not only opens a new path for the future research of CS/GO hydrogel scaffolds but also provides new ideas for the application of these scaffolds in promoting the formation of new blood vessels.

## Figures and Tables

**Table 1:** Sequences of primers for qRT-PCR.

Gene symbol	Forward primer (5′–3′)	Reverse primer (5′–3′)
*CD34*	AGACTGTGCAGTGATGTGGT	CCCTGGTACATTCGGGTCTG
*MMP9*	CCTGGGCAGATTCCAAACCT	GTACACGCGAGTGAAGGTGA
*VEGF*	GGCAAAAACGAAAGCGCAAG	GAGGCTCCAGGGCATTAGAC
*SDF-1*	TGCCCTTCAGATTGTAGCCC	GCGTCTGACCCTCTCACATC
*GAPDH*	GGAGTCCACTGGCGTCTTCA	GTCATGAGTCCTTCCACGATACC

**Table 2:** Physical properties of the CS/GO hydrogel scaffolds with different contents of GO.

Group	Tensile strength (MPa)	Modulus of elasticity (MPa)	Elongation at break (%)
CS	1.363 ± 0.367	0.829 ± 0.590	61.3
CS+0.1 wt.%GO	4.053 ± 0.526	6.863 ± 0.940	122.3
CS+0.5 wt.%GO	5.627 ± 0.343	12.460 ± 2.120	140.6
CS+1.0 wt.%GO	7.153 ± 0.383	22.026 ± 2.752	161.5

## Data Availability

Dr. Zhang, e-mail: summere0615@163.com, will provide the research data.
